# COVIDNearTerm: A simple method to forecast COVID-19 hospitalizations

**DOI:** 10.1017/cts.2022.389

**Published:** 2022-04-19

**Authors:** Adam B. Olshen, Ariadna Garcia, Kristopher I. Kapphahn, Yingjie Weng, Jason Vargo, John A. Pugliese, David Crow, Paul D. Wesson, George W. Rutherford, Mithat Gonen, Manisha Desai

**Affiliations:** 1 Department of Epidemiology and Biostatistics, University of California, San Francisco, CA, USA; 2 Helen Diller Family Comprehensive Cancer Center, University of California, San Francisco, CA, USA; 3 Quantitative Sciences Unit, Department of Medicine, Stanford University School of Medicine, Stanford, CA, USA; 4 California Department of Public Health, Sacramento, CA, USA; 5 Institute for Global Health Sciences, University of California, San Francisco, CA, USA; 6 Department of Epidemiology and Biostatistics, Memorial Sloan-Kettering Cancer Center, New York, NY, USA

**Keywords:** COVID-19, forecasting, hospitalization, prediction, SARS-CoV-2

## Abstract

**Introduction:**

COVID-19 has caused tremendous death and suffering since it first emerged in 2019. Soon after its emergence, models were developed to help predict the course of various disease metrics, and these models have been relied upon to help guide public health policy.

**Methods:**

Here we present a method called COVIDNearTerm to “forecast” hospitalizations in the short term, two to four weeks from the time of prediction. COVIDNearTerm is based on an autoregressive model and utilizes a parametric bootstrap approach to make predictions. It is easy to use as it requires only previous hospitalization data, and there is an open-source R package that implements the algorithm. We evaluated COVIDNearTerm on San Francisco Bay Area hospitalizations and compared it to models from the California COVID Assessment Tool (CalCAT).

**Results:**

We found that COVIDNearTerm predictions were more accurate than the CalCAT ensemble predictions for all comparisons and any CalCAT component for a majority of comparisons. For instance, at the county level our 14-day hospitalization median absolute percentage errors ranged from 16 to 36%. For those same comparisons, the CalCAT ensemble errors were between 30 and 59%.

**Conclusion:**

COVIDNearTerm is a simple and useful tool for predicting near-term COVID-19 hospitalizations.

## Introduction

Since first being identified in Wuhan, China in 2019, the SARS-CoV-2 virus and accompanying COVID-19 disease have had devastating consequences. As of May 1, 2022, and according to the website worldometers.info, the virus has caused over 6 million deaths worldwide and about a million deaths in the USA. At the peak of the pandemic, according to the CDC (https://covid.cdc.gov/covid-data-tracker/#hospitalizations), total hospitalizations have been nearly 150,000 on a single day in the USA. Among those who have recovered many have developed long-term health complications [1]. To combat the impact of the virus, starting in March and April of 2020 in the USA, various restrictions have been implemented. These restrictions have been maintained to various degrees in different communities. While restrictions were effective at slowing transmission [2], they have had a large impact on the economy [3], which has not been separate from the impact of the virus itself [4]. Predictive models, both long-term and short-term, were developed to help inform the level of restrictions and the amount of inpatient medical resources that would be needed.

Among long-term models, the first came from Imperial College [5]. This model made long-term predictions of healthcare demand that would result from potential interventions. Similar types of long-term predictions have been made throughout much of the pandemic by the Institute for Health Metrics and Evaluation [6] at the University of Washington.

In contrast, our model, COVIDNearTerm, is useful for the short term. Its particular use is for “forecasting,” which we define as making predictions two to four weeks into the future. We modeled the near term because the many sources of uncertainty deriving from a once-in-a-century pandemic make long-term predictions unrealistic. We focused on hospitalizations, because in the USA they reflect the number of severely ill cases in a population essentially independent of the amount of testing, since those sick enough will likely be admitted to a hospital. Accurately predicting hospitalizations is particularly important because hospital beds are a limited resource. Using only previous hospitalizations as input, which typically occur three to ten days after symptoms [7], our model’s effectiveness has been validated by comparing predicted hospitalizations to observed hospitalizations.

Major sources of variability in hospitalizations included recent trends, typical differences seen between days, and random error. To include these factors, we utilized an autoregressive model and made predictions using parametric bootstrap methods. This model can be helpful for answering questions about the future or past hospitalization trends relevant to planning and preparedness efforts. For example, the model could address the following questions regarding the future: What is the expected number of COVID-19 hospitalizations in a county in two weeks? What is the probability that the number of hospitalizations will exceed a pre-determined threshold, such as 100 in a county, any day in the next two weeks? A question involving the past could be: did a loosening of restrictions lead to an increase in hospitalizations? For example, four weeks after reopening restaurant dining, did hospitalizations increase more than would be expected given the previous trend?

We are not the first to model short-term hospitalizations. Early in the pandemic, Van Wees et al. [8] developed a Susceptible-Exposed-Infectious-Recovered (SEIR) model for this purpose. Such a model makes predictions by estimating the rate of transitions between these compartments. Perone [9] compared various time series models to predict near-term hospitalizations in the second wave in Italy. Goic et al. [10] used an ensemble method to predict short-term ICU hospitalizations.

The literature on COVID-19 prediction models has exploded since these early models were developed; for example, https://covid19forecasthub.org/ is currently tracking forecasts given by 45 models. Here we present relevant examples of each type of model we were able to identify. A critical review of the accuracy of pandemic modeling is given in Rosenfeld and Tibshirani [11] and a succinct discussion of the consequences of publicizing unreliable predictions in Divino et al. [12]. We focus on models with comparable goals – those predicting population-level outcomes, and exclude models predicting individual risk, even if population-level data were used to develop the model, as in Tanboga et al. [13]. We refer readers interested in those models to a systematic review [14].

Numerous predictions use algorithmic machine learning models, such as the long short-term memory recurrent neural network model; an example is Nikparvar et al. [15], which predicts county-level incidence. A similar model predicts hospitalization and death at a national level after vaccination [16]. Another group of models uses geographic and aggregate mobility data and mechanistic transmission models such as SEIR to predict population incidence and hospitalization rates [17,18,19]. In terms of short-term predictions, two recent novel approaches include utilizing small area data together with empirical models to make short-term predictions [20] and using auxiliary indicators [21].

Our model is novel in that it does not make any assumptions about the dynamics of the disease. It is a purely empirical approach and assumes that enough information is found in the current trends and previous trends of hospitalizations. Further, our model is pragmatic, relies on publicly available data, and is less computationally expensive than most models in the literature.

In June 2020, the state of California launched the California COVID Assessment Tool (CalCAT, https://calcat.covid19.ca.gov/cacovidmodels/). CalCAT gathered prediction models, mostly from independent academic research centers, to help predict the course of the COVID-19 pandemic in California in terms of key metrics. An underlying assumption was that by forming an ensemble, a model of models, predictions would be improved. Models include “nowcasts,” the current state of the pandemic, “forecasts,” as we have defined them, and “scenarios,” the long-term impacts of various policies. CalCAT forecasts were used to evaluate the accuracy of our model.

In this manuscript, we describe the COVIDNearTerm model for hospitalizations. We then assess how well our model predicted hospitalizations in the San Francisco Bay Area. Finally, we compare those results to those deriving from models included in CalCAT.

## Methods

Our modeling scheme, COVIDNearTerm, relies on an autoregressive model and makes predictions utilizing parametric bootstrap methods.

### Overview of the Model

**Fig. 1.**
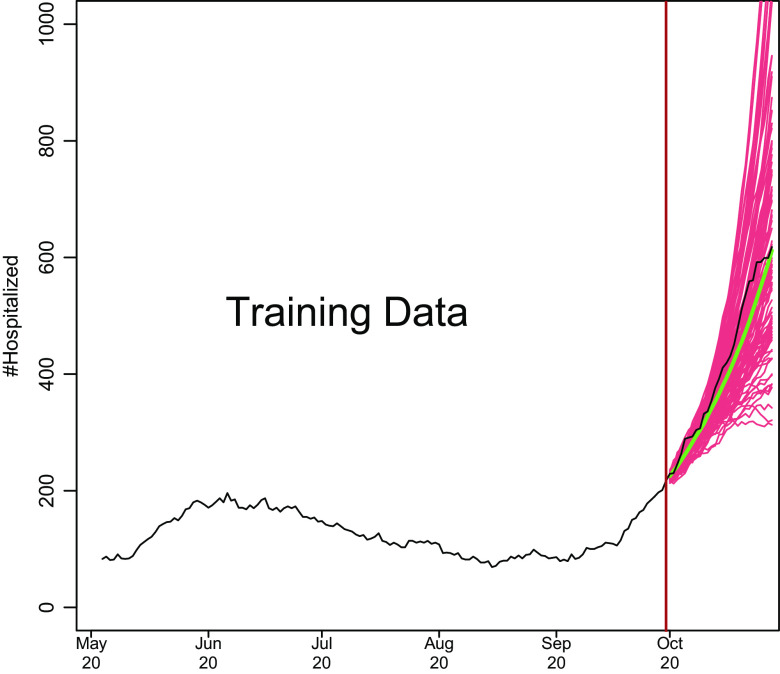
Demonstration of how COVIDNearTerm works. The black line depicts hospitalizations in Santa Clara County starting on May 4, 2020. The red lines represent 100 possible paths as predicted by the COVIDNearTerm model starting from October 1, 2020. Each path is for 28 days, and the prediction for a particular day, the median of the paths, is shown in green.

We show how model development works in Fig. 1. Based on a training set of 150 days starting on May 4, 2020, and running through September 30, 2020, we predict COVID-19 hospitalizations for the first 28 days of October 2020. This is based on real data from Santa Clara County, and we model the data exactly as described later. To make the hospitalization predictions, all we needed was a training set of hospitalizations and to specify two parameters: how many of the last days from the training set to use to specify the current trend and how heavily to weigh the very latest observations when estimating the trend. How much to dampen the trend is estimated from the training set as described below.

### The Basic Algorithm

Because COVID-19 cases, and thus hospitalizations, involve exponential growth or decline [22], like other infectious diseases [23], we developed a modeling approach able to fit such a pattern. Our basic model to account for this temporal dependency is
(1)

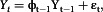

where *Y* represents observed hospitalizations, ε represents independent, zero-centered error, and *t* represents time in days. The basic idea is that the hospitalizations today are a multiple, represented by ϕ, of the hospitalizations yesterday plus random error. For predictions, the error is proportional to the value of *Y* as described below.

The training set is the first *T* days of data, and it is on the training data that the parameters of our model are estimated. These parameters are used later to make predictions. We start with a ϕ estimate of the form
(2)

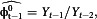

where the 0 superscript represents initial. To smooth daily trends while incorporating local trends, the final estimate of ϕ is a function of the past *W* days worth of data. The final form is
(3)

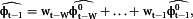

where the *w*s are weights. We considered three weighting schemes. The first was *equal* weights, *w*_*i*_ = 1/*W*, *i* = 1, …, *W*, and the second was triangular weights, 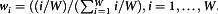 Triangular weights emphasize the most recent observations more heavily, and like equal weights, sum to 1. Note the subscript for ϕ of model (1) is *t* − 1, resulting in only data from previous times being used to predict the current time. For our two previous weighting schemes, when a new 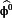 is added, the earliest one within the previous window of length *W* is eliminated. We introduce a third weighting scheme called *unweighted* that is the same as equal weighting except in one way. For unweighted updating, we randomly drop one 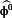 rather than the earliest one, which further damps down recent trends.

Fitting the model to the training set yields distributions of 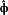 s and 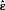 s. To predict future hospitalizations, we use these distributions and equation (1) to simulate paths of hospitalizations *H* days into the future, 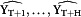 . We go from a measured hospitalization level *Y*_*T*_ to a predicted hospitalization level 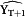 by simulating 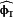 and 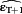 , as described below. We repeat this process iteratively to estimate 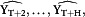 with each 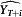 based on a previously simulated 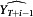 .

We repeat the simulation process *N* times. From the resulting *N* paths, we estimate hospitalizations *H* days into the future by the median of 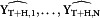 (or we could use other measures). Alternatively, we can calculate the maximum for each path, 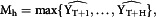 and estimate the probability of exceeding a trigger within *H* days as the proportion of *M*_*h*_s out of *N* that exceed the trigger.

We derive the estimate 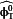 and thus 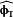 directly from training data including Y_T − 1_ and *Y*_*T*_. For 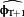 and beyond, we simulate based on our model. We simulate 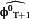 from a 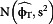 and add that to 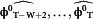 to estimate 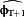 via equation (3). Here, 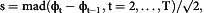 where mad is the scaled median absolute deviation estimate of the standard deviation. We estimate 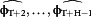 in a similar manner by updating 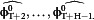


To simulate 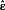 s, we first model the relationship between ε and *Y*. To do this, we use the locally weighted scatterplot smoothing method (LOWESS) to fit 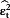 on *Y*_*t*_ for t = 1, …, T, since this provides an estimate of the variance of ε as a function of *Y* based on the training set. We simulate ε_T + 1_, …, ε_T + h_ from a 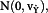 where 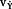 is estimated using the value of 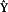 (minus the error term) and the LOWESS fit.

### Shrinking the Trend Estimate

Our experience with COVID-19 hospitalizations time series data is that trends tend to reverse themselves. Increasing trends come down while decreasing trends lead to future increases. Possible causes are that increasing trends lead to more careful behavior or a reversal of opening, while decreasing trends lead to less careful behavior and further opening. We therefore consider shrinking the trend towards ϕ = 1. This leads to the shrunken trend model
(4)

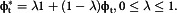




Here λ controls the amount of shrinkage, with λ = 0 imposing no shrinkage, and thus using the trend exactly as estimated, λ = 1 being a model with no trend, and the higher the λ the more the trend is attenuated. The model is utilized by substituting 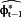 for 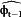 in model (1). The updating of 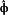 continues as if there was no shrinkage, while 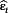 corresponds to the shrunken 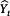 (again minus the error term).

To utilize the model, we need a method for estimating λ. We choose the 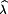 that best fits the training set *Y*s in the sense of the smallest sum of squared residuals. Alternatively, we could fix 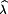 at some value based on previous experience.

### CalCAT Models

In the Results, we include data from nine CalCAT models that have been used for hospitalization forecasts at the county level in California. These models are categorized in Table 1 including references to preprints. Further technical details and code for these models can be accessed through CalCAT (https://calcat.covid19.ca.gov/cacovidmodels/). Seven of these models are of the SEIR variety. One model (Simple Growth) is exponential based on the current R-effective and case rate and uses historical hospital and intensive care unit admission rates. Another is a neural forecasting model (UCSB) that assumes predictions can be made by identifying similar patterns across regions from a few months prior. At the end of the data analysis period on May 1, 2021, only five models remained in CalCAT, four SEIR and Simple Growth. Simple Growth was utilized by CalCAT starting only on December 8, 2020.

**Table 1.** Models used by the California COVID Assessment Tool (CalCAT) for county-level hospitalization forecasts. Here "No" means the model was once used as part of CalCAT but was not at the end the analysis on May 1, 2021. SEIR stands for Susceptible-Exposed-Infectious-Recovered

Model	In CalCAT	Type of Model
COVID Act Now	No	SEIR
Columbia [24]	No	SEIR
JHU IDDG [25]	Yes	SEIR
LEMMA	Yes	SEIR
Simple Growth	Yes	Exponential using rate from R-effective
Stanford	Yes	SEIR
UCLA MLL	No	SEIR
UCSB [26]	No	Neural forecasting model
UCSD-COVIDReadi	No	SEIR

### Evaluating Prediction Errors

Because absolute errors in modeling would be expected to increase when hospitalizations increase, we focused on percentage error. More specifically, we utilized the median absolute percentage error (MedAPE).

The MedAPE is
(5)

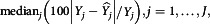

where *J* is the number of days for which we provided predictions. For some predictions, we also report the 25th and 75th percentiles of absolute prediction errors, which we put in brackets. Prediction accuracy was compared between models using the MedAPE. As a secondary assessment of the models, we estimated the Pearson correlation between true and predicted hospitalizations.

### Data, Code, and Utilization

The historical data in this manuscript were downloaded on May 11, 2021, from CalCAT. These data are of the aggregate number of COVID hospitalizations for each county for each day. Importantly, there are no longitudinal patient-level data, so there are no data on when a patient entered the hospital, when they tested positive for COVID-19, and whether they are in the hospital because of COVID-19 or for another reason. Further, there is also no demographic information about the hospitalized patients.

Code is available at https://github.com/olshena/COVIDNearTerm/. It is in the form of an R package called COVIDNearTerm, and we used version 1.0 for the analysis in this manuscript. Our predictions for all California counties can now be found as part of the CalCAT county forecasts at https://calcat.covid19.ca.gov/cacovidmodels/. While COVIDNearTerm is currently part of the CalCAT ensemble, it was not during the time for which there are comparisons in the manuscript.

The analysis and figures in this manuscript can be reproduced using the file https://github.com/olshena/COVIDNearTerm/Manuscript/reproduce.zip.

## Results

We assessed COVIDNearTerm by comparing it to models in CalCAT. We focused on hospitalization data from the six inner Bay Area counties. They are, ordered by size, Marin (0.3 million people), San Mateo (0.8), San Francisco (0.9), Contra Costa (1.2), Alameda (1.7), and Santa Clara (1.9). We made predictions based on all the training data comprised of hospitalizations reported from before that day. We made predictions for 14 days, 21 days, and 28 days and made predictions based on 1000 simulations. We started training models with data from May 4, 2020, as we wanted data collection to stabilize before we started, and made our first predictions utilizing data up to June 14, 2020. This gave the first 14-day predictions for June 28, the first 21-day predictions for July 5, and the first 28-day predictions for July 12. We made predictions for every day through May 1, 2021.

**Fig. 2.**
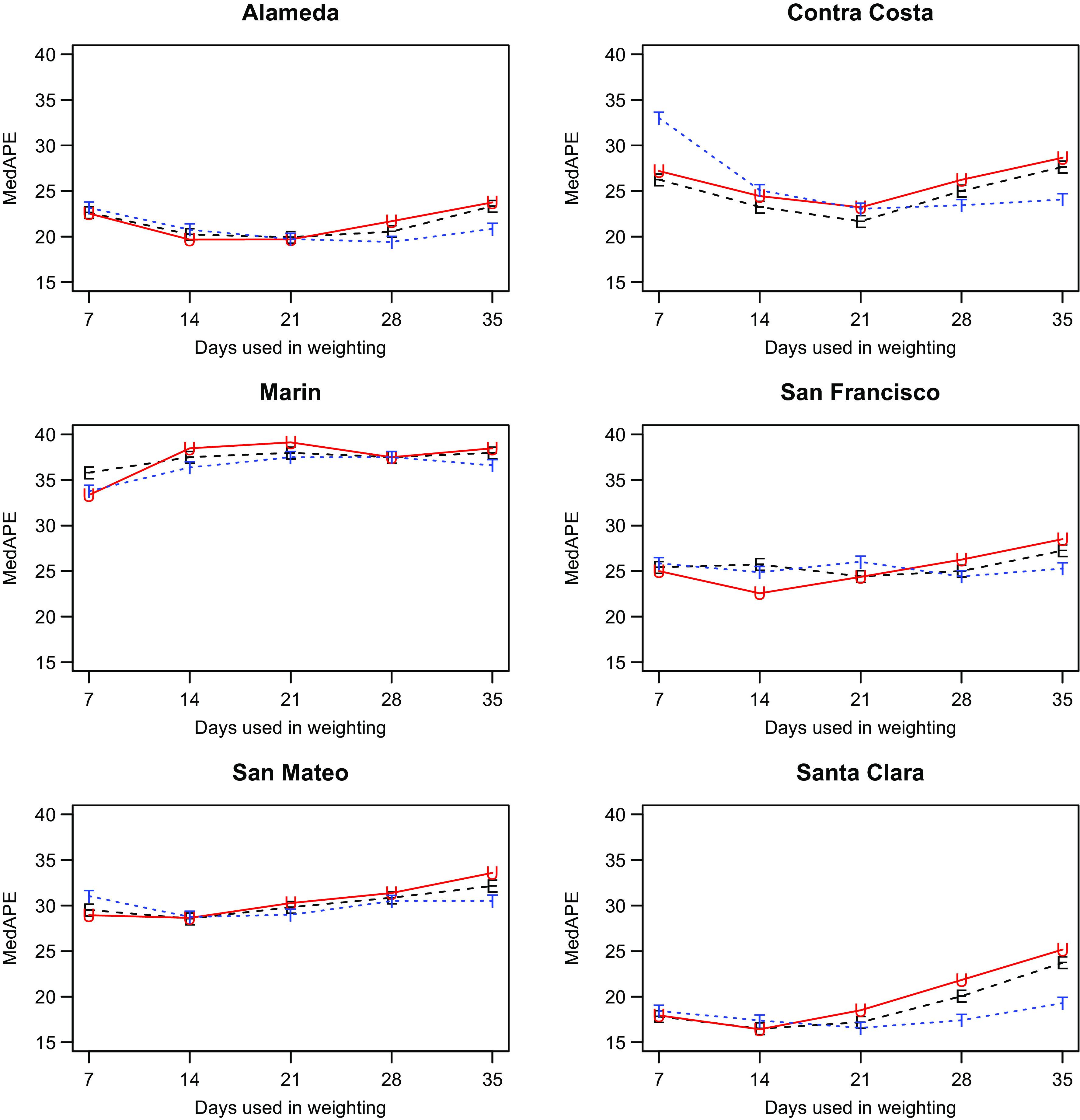
Median absolute percentage error (MedAPE) by county as a function of days used in weighting for 14-day predictions. The symbol E is for equal (black), U is for unweighted (red) and T is for triangular (blue).

We discuss only our models with shrinkage as they had better performance (data not shown). That left us with two parameters to consider: the weighting method and the number of days, *W*, utilized for weighting. As shown in Fig. 2, which has the MedAPE for 14-day predictions, the weighting method had little impact. For *W*, 14 or 21 tended to do best across counties. Overall, the unweighted method with W = 14 gave the smallest MedAPE for Santa Clara (16% [7%,28%]) and San Francisco (23% [10%,45%]), and no other combination was best for more than one other county. Also, across counties, this combination had the lowest average MedAPE of 25.0% [11.4%,42.0%], with the next closest being 25.3% [11.7%,41.8%] for equal weighting with W = 14. Therefore, the unweighted method with W = 14 was used for all further comparisons.

**Fig. 3.**
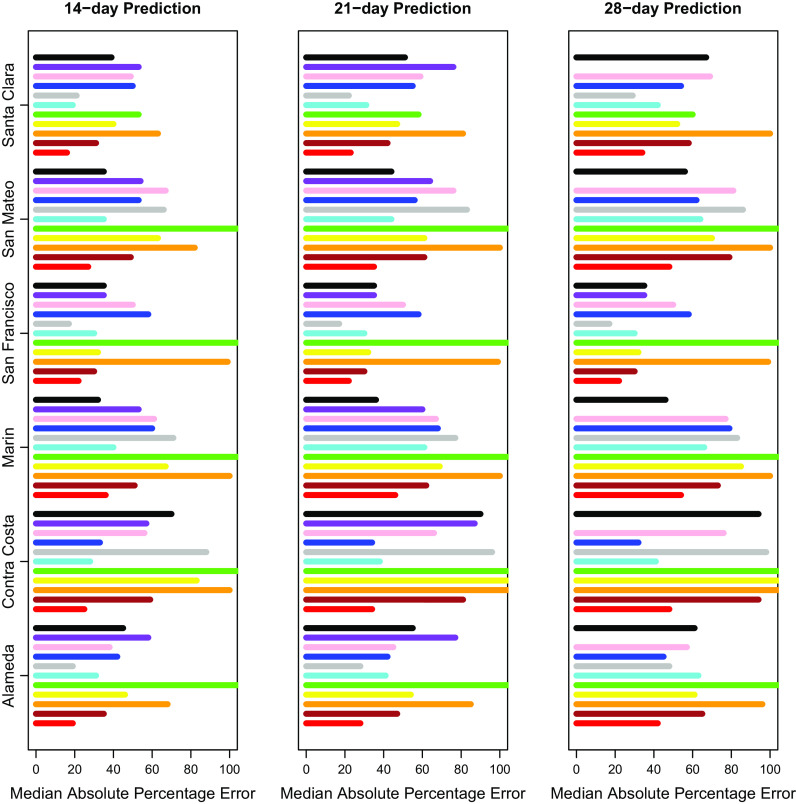
Median absolute percentage error by county for 14 days, 21 days and 28 days. Here 14 days is on the left, 21 days is in the middle, and 28 days is on the right. The models from bottom to top are COVIDNearterm (red), CalCAT Ensemble (brown), COVID Act Now (orange), Columbia (yellow), JHU IDDG (green), LEMMA (cyan), Simple Growth (gray), Stanford (blue), UCLA MLL (pink), UCSB (purple), and UCSD-COVIDReadi (black).

After selecting our modeling approach, we did further comparisons only on days where CalCAT had ensemble model predictions, which lowered the number of days with predictions from 308 to 283 but changed our prediction errors only slightly. Results across counties for 14-day, 21-day, and 28-day predictions can be found in Fig. 3 and Table 2. The MedAPEs ranged from 16 % -36% for 14-day predictions, 23 % -46% for 21-day predictions, and 34 % -54% for 28-day predictions. All the lowest errors were for Santa Clara (the most populous county), and all the highest errors were for Marin (the least populous county).

**Table 2.** Median absolute percentage error by county for COVIDNearTerm and all California COVID Assessment Tool (CalCAT) models at 14 days, 21 days and 28 days. We use the abbreviations AL (Alameda), CC (Contra Costa), MA (Marin), SF (San Francisco), SM (San Mateo) and SC (Santa Clara). The models are discussed in Table 1. Note that COVID Act Now often gave median absolute percentage errors of 100 because the predicted hospitalizations were zero

Model	AL	CC	MA	SF	SM	SC
COVIDNearTerm	19,28,42	25,34,48	36,46,54	22,38,45	27,35,48	16,23,34
CalCAT Ensemble	35,47,65	59,81,94	51,62,73	30,46,58	49,61,79	31,42,58
COVID Act Now	68,85,96	100,143,170	100,100,100	99,100,100	82,100,100	63,81,100
Columbia	46,54,61	83,104,109	67,69,85	32,46,53	63,61,70	40,47,52
JHU IDDG	144,178,202	237,301,300	320,378,382	127,162,180	231,301,348	53,58,60
LEMMA	31,41,63	28,38,41	40,61,66	30,43,57	35,44,64	19,31,42
Simple Growth	19,28,48	88,96,98	71,77,83	17,19,35	66,83,86	21,22,29
Stanford	42,42,45	33,34,32	60,68,79	58,64,78	53,56,62	50,55,54
UCLA MLL	38,45,57	56,66,76	61,67,77	50,63,74	67,76,81	49,59,69
UCSB	58,77,NA	57,87,NA	53,60,NA	35,43,NA	54,64,NA	53,76,NA
UCSD-COVIDReadi	45,55,61	70,90,94	32,36,46	35,36,40	35,44,56	39,51,67

We compared our results to those from the CalCAT tool. The comparisons based on the MedAPE were to both the CalCAT Ensemble and the individual CalCAT components. COVIDNearTerm was more accurate than the Ensemble for all counties at 14, 21, and 28 days. For instance, in Santa Clara County, the COVIDNearTerm MedAPEs were 16% [7%,26%], 23% [10%,47%], and 34% [15%,67%] at 14, 21, and 28 days compared to 31% [14%,49%], 42% [24%,65%], and 58% [31%,84%] for the Ensemble. For Alameda, the MedAPEs for the same comparisons were 19% [11%,30%], 28% [13%,45%], and 42% [16%,65%] versus 35% [17%,59%], 47% [26%,78%], and 65% [46%,102%].

**Fig. 4.**
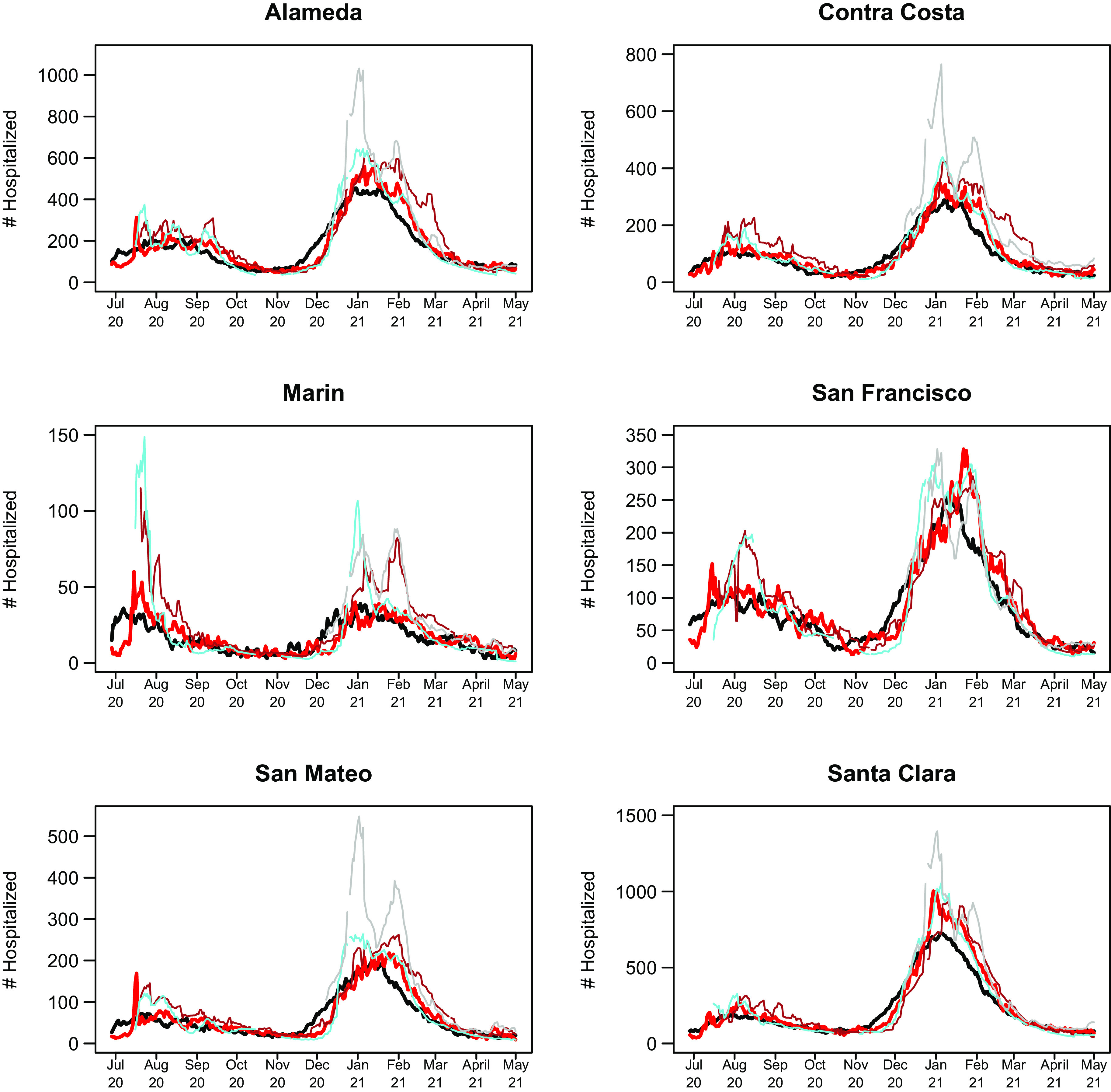
14-day predictions for multiple methods. The lines are for truth (black), COVIDNearTerm (red), CalCAT Ensemble (brown), LEMMA (cyan), and Simple Growth (gray). Note that Simple Growth was utilized by CalCAT starting only on December 8, 2020.

The 14-day predictions for COVIDNearTerm, the CalCAT Ensemble, and two promising methods, LEMMA and Simple Growth, can be seen in Fig. 4. Qualitatively, COVIDNearTerm had better predictive performance than the Ensemble in part because it more quickly predicted a decrease after the January 2021 peak. Differences among COVIDNearTerm and LEMMA and Simple Growth were less systematic.

At the individual model level, COVIDNearTerm was most accurate except where stated. For Alameda, Simple Growth was equally accurate for 14-day predictions (MedAPE = 19%) and 21-day predictions (MedAPE = 28%). For Contra Costa, Stanford was equally accurate for 21-day predictions (MedAPE = 34%) and more accurate for 28-day predictions, with MedAPE = 32% versus 48% for COVIDNearTerm. LEMMA was also more accurate for 28-day predictions with MedAPE = 41%. UCSD-COVIDReadi was more accurate for Marin at 14 21 and 28 days, with MedAPEs of 32, 36, and 46% versus 36, 46, and 54% for COVIDNearTerm. For San Francisco, Simple Growth was more accurate for all three with MedAPEs of 17, 19, and 35% versus 22, 38, and 45% for COVIDNearTerm. UCSD-COVIDReadi was also more accurate for 21-day (MedAPE = 36%) and 28-day (MedAPE = 40%) predictions. For Santa Clara, Simple Growth was more accurate for 21-day and 28-day predictions, with MedAPEs of 22 and 29% versus 23 and 34% for COVIDNearTerm.

Overall, COVIDNearTerm was most accurate or equal to most accurate for 10 of 18 comparisons and four of six 14-day comparisons, all but Marin and San Francisco. It was also never worse than the third most accurate model for any comparison.

To confirm that the strong results for COVIDNearTerm were not dependent on our preferred measure, MedAPE, we also evaluated accuracy using the Pearson correlation between true hospitalizations and model-based hospitalizations. We focused on the two-week results. Using this measure, COVIDNearTerm was the second most accurate model with LEMMA the most accurate. For Alameda and Santa Clara Counties, COVIDNearTerm and LEMMA were most accurate with correlations of 0.88 and 0.95, respectively. For Contra Costa, LEMMA was most accurate with a correlation of 0.90 and COVIDNearTerm was the second most accurate with a correlation of 0.88. For San Francisco, Simple Growth was most accurate with a correlation of 0.83 followed by COVIDNearTerm and LEMMA at 0.82. For San Mateo, UCSD was most accurate with a correlation of 0.91, Simple Growth had a correlation of 0.83, and LEMMA and COVIDNearTerm had correlations of 0.82. Finally, for Marin, the least populated county, Simple Growth was best with a correlation of 0.67, while COVIDNearTerm was only the sixth best with a correlation of 0.46, lower than the ensemble, which had a correlation of 0.60. Thus, COVIDNearTerm was the most accurate or close to the most accurate for all but one county.

## Discussion

We developed an autoregressive model called COVIDNearTerm for forecasting hospitalizations two to four weeks out. It has the virtue of only requiring previous hospitalization data, so it is widely applicable. When applied to CalCAT data, its predictions were more accurate than the CalCAT ensemble model for all comparisons and more accurate than any other CalCAT model for most comparisons.

As mentioned, COVIDNearTerm uses only past hospitalizations to predict future hospitalizations. This approach would be disadvantageous if the predictions were inferior to those based on other factors such as testing, community transmission, and the percentage of people who have already been infected. We have demonstrated, however, that COVIDNearTerm is as accurate or more accurate than models that include such covariates. Therefore, we were able to do more with less.

There are a few caveats to our modeling. Our comparisons to the models in COVIDNearTerm may have been slightly biased to favor COVIDNearTerm. First, we know the publication date of the CalCAT component models, which is when the predictions appeared on the CalCAT site, but not the actual date the predictions were made. We assume that the publication date was close to the prediction date, and the modelers did have the option of making frequent prediction updates. Second, we looked at the data retrospectively with COVIDNearTerm. Therefore, we had the most updated data on actual hospitalizations, which might be slightly different than when the other models made their predictions. But since we did not start our comparisons until June 2020, most data issues should have been resolved. Overall, we believe the performance metrics we have presented are valid.

The largest errors in predictions for our time of study, for COVIDNearTerm and other models, were between December 2020 and February 2021. This is the same period where there was the biggest change in hospitalizations, first an increase and then a decrease. It makes sense that the models under consideration had the greatest prediction error during periods of rapid change.

We saw a modest impact on hospitalizations based on the day of the week, which was much less than the impact of day of the week on cases (data not shown). If adjusting for day of the week is desired, we suggest making the adjustment outside of COVIDNearTerm. By making predictions 14, 21, or 28 days in the future, we mostly avoided the day of the week problem. We also did not address the modest impact of holidays.

One weakness of COVIDNearTerm is that its predictions generally monotonically increase or decrease over time. We might believe that hospitalizations will, for instance, decrease four weeks out based on a recent decrease in cases or test positivity, even if this reduction has not yet been seen in hospitalizations. For this situation, the next generation of COVIDNearTerm could include an adjustment for covariates. How to adjust, however, is not straightforward, as the relationships among covariates and hospitalizations can change over time (data not shown).

There are a couple additional details that should be understood before using COVIDNearTerm. First, our model is appropriate for outcomes that exponentially increase or decrease and that start one time period (such as day) where they ended the previous time period. It would be less appropriate for outcomes where measurements are independently measured in each time period, such as new hospitalizations. Second, users of COVIDNearTerm may want to optimize over weighting schemes and number of days that go into weighting, that is, *W*.

We have shown that a simple hospitalization modeling strategy was as effective as anything else used by CalCAT at a time of great uncertainty. We believe that the less you know at the time of modeling the more a simple approach makes sense. Further, our modeling strategy can be extended to other contexts. For example, it may be helpful in some places in the world where related covariates are missing or not measured accurately. It might also be applicable to other pandemics, including ones resulting from new SARS-CoV-2 variants.
